# Notes on Biliary and Intestinal Sand

**Published:** 1910-06

**Authors:** George Parker

**Affiliations:** Physician to the Bristol General Hospital.


### NOTES ON BILIARY AND INTESTINAL SAND.

BY

GEORGE PARKER, M.D.,

*Physician to the Bristol General Hospital.*

---

I HAVE recently attended two patients both of whom passed gall-stones, and both of whom noticed a quantity of fine gritty sand in their stools for several days and even weeks during the time that the stones were passing. The stones were of the ordinary type, facetted, varying in size from a hemp seed to a pea, and composed almost entirely of cholesterin. I learnt that several tablespoonfuls of sand were passed in one case, but much less in the other. There was no pain or other special symptom attending its passage. The clinical history is briefly as follows:—

Dr. A. is a medical man of middle age, engaged in active practice, and has always enjoyed good health. In October, 1909, he had an attack of biliary colic, which lasted an hour or two, and was accompanied by vomiting. In December another attack came on one Sunday evening while he was at church, and a slight one followed on the Tuesday. In January the colic returned with slight jaundice, vomiting and some pyrexia, and in February a final attack, after which three calculi the size of a pea and others smaller were passed. The calculi were somewhat soft, and broke when handled. During this last attack the sand was noticed sinking slowly to the bottom when the stools were mixed with water. It disappeared about a week after the last stone was found. There has never been any colitis, and the bowels have always been regular with a slight tendency to looseness. The patient is now, I am glad to say, in perfect health.

Mr. B. is an elderly gentleman who formerly led a very active life. He is a total abstainer and a moderate eater, though fond of fruit. The bowels tended to be constipated, and the patient's general health has been excellent. He first felt the biliary colic at Christmas, 1908. In the July following he had four more attacks, and one in November. In December a slight one occurred and was followed by jaundice. At the beginning of January, 1910, in a fresh attack of pain his temperature rose to 104°, with vomiting, rigors and jaundice and considerable prostration. On the 14th nausea was complained of, then rigors and vomiting, during which he brought up two calculi. During the next fortnight seventy-four stones, with a large quantity of sand, were found in the stools. The sand was at first light in colour and coarse or gravelly, but it was afterwards dark and finer. Slight colic with jaundice recurred from time to time for six weeks, till in all 112 stones were passed. The sand continued to appear most of the time. The tenderness over the region of the gall-bladder, which had been present throughout, now disappeared. Since then the patient has had no further relapse or any other symptoms.

The composition of the sand in both these cases rather surprised me. It would be natural to suppose that sand passed at the time of the expulsion of gall-stones in otherwise healthy persons, and apparently only at that time would be composed of substances from the biliary passages; but this sand contains neither cholesterin, bilirubin, or bilihumin-calcium. These substances are the material of true biliary sand, and are sometimes seen in a finely granular condition in or near the bile passages. Biliary sand, indeed, is often spoken of, and sometimes referred to as being passed per anum, but I have failed to find any detailed account or analysis of it when thus passed. Naunyn, too, is quite sceptical about the "gall sand" of various writers, and thinks it unlikely that such biliary sand could pass down the intestine without being broken up. (*Cholelithiasis*, p. 85.)

He goes on to tell a story of someone who undertook to send him specimens from six different patients, but the sand turned out to be only vegetable debris. Our specimens, on the other hand, show no vegetable cells or characteristic structures, and the inorganic matter in the portion analysed amounts to 20.7 per cent., and proves to be calcium phosphate with traces of sulphate and carbonate. (Drs. Roberts and E. R. Short.)

Dr. E. Emrys-Roberts reports as follows :—

"The appearances and reactions are similar in both cases. The sand consists of irregular, amorphous, reddish-brown masses of a pronounced degree of hardness, which when washed and dried in the air appeared as a fine, gritty, deep reddish-brown sand. Under low power there was no evidence of crystalline formation, the outlines were irregular.

"When extracted with ether there was no deposit of cholesterin crystals, and the substance gave none of the reactions of altered blood. When washed and treated in the cold with dilute caustic soda a yellowish solution was obtained, but this solution did not give Gmelin's reaction for bile-pigment with fuming nitric acid, nor yet the blue colour which this reagent produces in the presence of bilihumin. The test for bilirubin with dilute tincture of iodine was also negative. When treated with concentrated hydrochloric acid under a cover-glass bubbles of gas were seen to be evolved, but solubility was extremely slow. This reaction took place with both concentrated sulphuric acid and strong nitric acid, but not with glacial acetic acid. In the organic residue no evidence was seen of a cellular basis.

"When the organic constituents were removed by combustion the inorganic residue appeared of a pearly, bluish-white colour, the particles being fragile, not altered in shape, but probably in size. The difficulty of being certain on this point is obvious.

"A portion of the original substance was treated with acetic acid, and the filtrate shaken up with ether. The latter did not become tinted a pink shade but remained colourless."

The substance, then, is clearly not the same as the hard, granular, blackish material composed of bilirubin or bilihumin-calcium which forms true biliary sand, but it corresponds very nearly to one type of intestinal sand as described by various writers.

Intestinal sand of any kind is rare, but most writers hold that there are two distinct forms. The first has a vegetable basis, due to sclerenchymatous particles in the fruit of the pear, the banana or the pea. On examination, there are sometimes seen to be woody cells, in the thick walls of which are channels running from the cavity to the surface. The inorganic matter is usually only 2 or 3 per cent. Marcet wrote about this pear sand as long ago as 1817. Robin and Laboulbène[1] in 1873, in reporting six cases, showed that in one at least the substances consisted of definite vegetable cells with an incrustation of lime salts. Shattock[2] and others have reported similar instances. In a case of Thomson's[3] the sand could be produced at will by giving pears in the food, and it disappeared if they were left off.

The second form of intestinal sand has no vegetable skeleton, and contains a much greater quantity of inorganic matter. Thus Mathieu and Richaud[4] give two instances with about 60 per cent. of lime salts. Thomson and Ferguson[5] one of 71, Monjour[6] one with 70 per cent. of lime and magnesia, and one of Dieulafoy's[7] cases had a similar composition. Duckworth and Garrod[8] found 61 per cent. of calcium phosphate, Marquez[9] a specimen as low as 28. We need not include the minute crystalline bodies mentioned by Schönlein[10] and Bellingham,[11] but generally this type of sand is formed of hard grains made up of calcareous salts and more or less homogeneous organic matter. Eichorst thought the latter was composed of crystals of fatty acids in one case. Bedford[12] says that the percentage of inorganic matter is usually from 28 to 70 per cent.

Duckworth's description of the sand shows many points of resemblance to that passed by my patients. It was, he says, of a deep brown with a mixture of pale particles. The granules were of various shapes, none of them crystalline or cellular. Treated with an acid, the hard sand gave off bubbles, and left an organic base soluble in alkalies. A pink colouring matter could be extracted, and the spectrum of urobilin was detected. There was very little unaltered bile pigment. He concludes that it is formed in a part of the intestine where the bile pigment is already largely broken up and changed to urobilin. As the large intestine appears to excrete a considerable amount of calcium and magnesium, the inorganic matter may thus be independent of the food.

Now the clinical history of patients who pass this type of sand is frequently that of mucous colitis, or of an intractable diarrhœa without mucous casts of the bowel. Sometimes a gouty history is recorded, and French writers have claimed a special lithiatic diathesis; but in some, as in a case of Dietz[13] in 1901, there is no history of either diarrhœa or gout, and occasionally there has been constipation. Duckworth says two-thirds of the cases have been in women, and the average age is 35 years. Dieulafoy and others have found it in children who suffered from constipation, and in them, as in adults, the discharge was often preceded by attacks of colic. Emerson[14] thinks it is the result of a secretory neurosis of the intestine. It occurs in neurasthenic patients, and frequently accompanies membranous colitis. The stools are often preceded by an hour of severe pain.

Owen Williams[15] has shown that this sand, besides the inorganic basis, contains fatty acids and soap, the fats being of the stearic or palmitic type, and he notes the prevalence in his cases of colicky pains and various forms of colitis. He thinks that it is due to some abnormality in the digestion of these fats, and the production of calcium salts of saturated fatty acids. He has been able to show that an isolated loop of bowel or the appendix will sometimes excrete fat of this type, and even form concretions of it in the appendix. In one patient who passed this intestinal sand many very small, brownish-red granules were found actually in the bile passages and the neighbouring parts of the intestine when he was operated upon, and these granules also contained fat. Moreover, Williams found that the excretion of sand could be prevented by giving olive oil in place of ordinary fat in the food. His view, therefore, of true intestinal sand is practically that of Nothnagel, *i.e.* that it is due to a lithogenic catarrh, and he confirms the observations of Eichorst and Roesser[16] that the sand contains fat. In the case of one of my patients an analysis by Mr. O. C. M. Davis, B.Sc., gave the following result :—

The specimen was washed, dried and extracted with ether in a Soxhlet's apparatus, and gave 7.1 per cent of fat. The residue, after extraction of fat, was treated with hydrochloric acid, taken to dryness, and again extracted with ether, giving 4.1 per cent. extract, which consisted partly of free fatty acid, as far as was possible to judge from the small quantity available.

Myer and Cook [17] take a different view, denying the distinction of the two types. They think that both forms have a vegetable nucleus, and when this is retained long in the bowel it is coated with lime. To explain the seemingly frequent combination with mucous colitis and dysentery, they point out that it is chiefly in such cases that the stools are examined. Their own patient had chronic constipation. The sand proved to be due to bananas, and could be produced at will by giving one or two bananas a day. They find that the cells of the milk tubes in this fruit are arranged in rows, and contain balls of resin with a fluid rich in tannin. These balls when hardened formed the sand, as Edes and Bates [18] had previously shown in other cases of banana eaters.

In one of my patients I have evidence that no bananas were eaten at the time of the excretion of sand, and that very few were taken by the other person.

If we are inclined to accept Myer and Cook's view of this external origin of all intestinal sand, why do not lime salts commonly incrust various nuclei in the fæces in ordinary constipation ? The stools are certainly examined far too often for it to be passed unobserved. I think that the weight of evidence is altogether in favour of the true sand generally occurring together with some kind of intestinal catarrh, and the only difficulty is to understand why it is so rare in cholelithiasis, for in that condition there is certainly a catarrhal state not confined to the bile passages.

We know that an excretion of lime takes place from their mucous lining, and is a factor in the formation of the bilirubin calcium nucleus of most gall-stones, and that this excretion is probably increased in catarrhal states, and renders possible the deposition of cholesterin. It may be that sand is only formed, as Williams suggests, when there is also a perverted digestion of fats leading to the formation of the organic matrix or a calcium salt of certain fatty acids.

REFERENCES.

1 Laboulbène, *Bull. Acad. Med. Paris*, 1873, xxii. 641.
2 Shattock, *Tr. Path. Soc. Lond.*, 1897, xlviii 124.
3 Thomson and Ferguson, *J. Path. and Bacteriol*, 1900, vi 334.

[4] Mathieu et Richaud, *Soc. Med. Hop. Paris*, May 22, 1896.
[5] Thomson and Ferguson, *Loc. cit.*
[6] Mongour, *Compt. rend. Soc. de Biol.*, 1896, p. 203.
[7] Dieulafoy, *Bull. Acad. Med. Paris*, 1897, xxxvii, 287.
[8] Duckworth and Garrod, *Med. -Chir. Tr.*, 1901, lxxxiv, 389.
[9] Marquez, *Assoc. franç. pour l'Avanc. d. Sc.*, 1879, 878.
[10] Schönlein, *Arch. f. Anat., Phys. u. wissensch. Med.*, 1836, 258.
[11] Bellingham, *Dublin J. M. Sc.*, 1838, xiv, 278.
[12] Bedford, *Lancet*, 1902, ii, 215.
[13] Dietz, *Deutsche Arch. f. klin. Med.*, 1901, lxx, 365.
[14] Emerson, *Clinical Diagnosis*, 1906, p. 371.
[15] Owen Williams, *Bio.-Chem. J.*, 1907, ii, 9, 395.
[16] *Deutsche Arch. f. klin. Med.*, lxviii, Hft. 1 and 2.
[17] Myer and Cook, *Am. J. M. Sc.*, 1909, cxxxvii, 383.
[18] Bates, *Am. J. M. Sc.*, 1887, xciii, 440.

### NOTES ON A CASE OF OXYCEPHALY.

BY

G. HELY-HUTCHINSON ALMOND, M.B., M.A.Oxon.

